# Mendelian randomization prioritizes abdominal adiposity as an independent causal factor for liver fat accumulation and cardiometabolic diseases

**DOI:** 10.1038/s43856-022-00196-3

**Published:** 2022-10-13

**Authors:** Eloi Gagnon, William Pelletier, Émilie Gobeil, Jérôme Bourgault, Hasanga D. Manikpurage, Ina Maltais-Payette, Erik Abner, Nele Taba, Tõnu Esko, Patricia L. Mitchell, Nooshin Ghodsian, Jean-Pierre Després, Marie-Claude Vohl, André Tchernof, Sébastien Thériault, Benoit J. Arsenault

**Affiliations:** 1 Centre de recherche de l’Institut universitaire de cardiologie et de pneumologie de Québec, Québec, QC Canada; 2 School of Nutrition, Université Laval, Québec, QC Canada; 3 Estonian Genome Center, Institute of Genomics, University of Tartu, Riia 23b, Tartu, 51010 Estonia; 4 Institute of Molecular and Cell Biology, University of Tartu, Riia 23, Tartu, 51,010 Estonia; 5 VITAM – Centre de recherche en santé durable, Université Laval, Québec, QC Canada; 6 Centre NUTRISS, Institut sur la nutrition et les aliments fonctionnels, Université Laval, Québec, QC Canada; 7 Department of Molecular Biology, Medical Biochemistry and Pathology, Faculty of Medicine, Université Laval, Québec, QC Canada; 8 Department of Medicine, Faculty of Medicine, Université Laval, Québec, QC Canada

**Keywords:** Heart failure, Type 2 diabetes

## Abstract

**Background:**

Observational studies have linked adiposity and especially abdominal adiposity to liver fat accumulation and non-alcoholic fatty liver disease. These traits are also associated with type 2 diabetes and coronary artery disease but the causal factor(s) underlying these associations remain unexplored.

**Methods:**

We used a multivariable Mendelian randomization study design to determine whether body mass index and waist circumference were causally associated with non-alcoholic fatty liver disease using publicly available genome-wide association study summary statistics of the UK Biobank (*n* = 461,460) and of non-alcoholic fatty liver disease (8434 cases and 770,180 control). A multivariable Mendelian randomization study design was also used to determine the respective causal contributions of waist circumference and liver fat (*n* = 32,858) to type 2 diabetes and coronary artery disease.

**Results:**

Using multivariable Mendelian randomization we show that waist circumference increase non-alcoholic fatty liver disease risk even when accounting for body mass index (odd ratio per 1-standard deviation increase = 2.35 95% CI = 1.31–4.22, *p* = 4.2e−03), but body mass index does not increase non-alcoholic fatty liver disease risk when accounting for waist circumference (0.86 95% CI = 0.54–1.38, *p* = 5.4e−01). In multivariable Mendelian randomization analyses accounting for liver fat, waist circumference remains strongly associated with both type 2 diabetes (3.27 95% CI = 2.89–3.69, *p* = 3.8e−80) and coronary artery disease (1.66 95% CI = 1.54–1.8, *p* = 3.4e−37).

**Conclusions:**

These results identify waist circumference as a strong, independent, and causal contributor to non-alcoholic fatty liver disease, type 2 diabetes and coronary artery disease, thereby highlighting the importance of assessing body fat distribution for the prediction and prevention of cardiometabolic diseases.

## Plain language summary

Non-alcoholic fatty liver diseases (NAFLD) affects around 25% of adults worldwide. NAFLD occurs when fat accumulates in the liver. Individuals with an elevated body weight are at higher risk of accumulating liver fat and developing cardiometabolic diseases such as NAFLD. Here, we show that people who inherit an increased likelihood to store fat inside the abdomen are at higher risk of liver fat accumulation and cardiometabolic diseases such as NAFLD, type 2 diabetes, and coronary artery disease. Conversely, individuals who store fat outside the abdomen may be at lower risk of liver fat accumulation and NAFLD. Based on these results, we conclude that the amount of fat in the abdomen rather than total body weight may be a key risk factor for cardiometabolic diseases.

## Abstract (web summary)

Gagnon et al. perform a Mendelian randomization study to investigate whether excess liver fat underpins adiposity’s effect on cardiometabolic disease risk. They show that abdominal adiposity strongly increases disease risk independently of liver fat, suggesting that targeting liver fat alone may not be sufficient to prevent cardiometabolic disease.

## Introduction

Non-alcoholic fatty liver disease (NAFLD) is characterized by hepatic lipid accumulation ranging from steatosis (>5% of liver weight is lipids) to non-alcoholic steatohepatitis (NASH, presence of inflammation)^1^. Although liver steatosis may be relatively benign in most cases, more severe forms of NAFLD such as NASH and hepatic fibrosis can lead to liver cirrhosis and hepatocellular carcinoma. Approximately 25% of the adult population globally is affected by NAFLD with the prevalence rapidly increasing and potentially becoming the leading cause of liver failure in the United States by 2025^2,3^. Adiposity and body fat distribution are closely linked with NAFLD^4^. In observational studies such as the INSPIRE ME study, a large international imaging study using computed tomography, waist circumference was closely associated with liver fat accumulation independently of body mass index (BMI)^5^.

Studies have also shown that both liver fat accumulation/NAFLD and waist circumference are associated with CAD and T2D^6–9^. However, whether liver fat accumulation is a causal factor of CAD and T2D remains to be elucidated and, more importantly, whether or not agents aimed at targeting NAFLD will ultimately decrease the risk of either T2D or CAD is unknown. In a previous investigation, we showed a strong genetic correlation between NAFLD, waist circumference, T2D, and CAD^10^. However, little is known about the directionality of these relations and whether NAFLD lies in the causal pathway linking abdominal adiposity and T2D/CAD.

In order to gain insight about the causality and directionality of these associations, causal inference methods such as Mendelian randomization (MR) have been developed^11^. MR uses genetic variants (which are randomly distributed at meiosis) such as single-nucleotide polymorphisms (SNPs), as instruments to infer causality. This method is in many ways comparable to a randomized control trial in which participants are naturally randomized based on the presence or absence of genetic variants that influence traits of interest^11^. In previous MR studies, a body fat distribution pattern consistent with low peripheral/subcutaneous fat accumulation and high intra-abdominal fat accumulation as estimated by the waist-to-hip ratio (WHR) adjusted for BMI was strongly associated with T2D and CAD^12,13^. However, we do not know if similar associations exist for NAFLD.

Extensions of the MR design, such as bidirectional MR and multivariable MR (MVMR), help in clarifying causal relations. Bidirectional MR refers to an analysis where both traits are alternately evaluated as exposure and outcome. This method has the potential to remove reverse causation bias by asserting the directionality of the relationship^14^. Multivariable MR can be used when multiple genetic variants are associated with two or more exposures. It conditions the effects of the SNPs of each exposure together to assess the effect of each exposure independently on the outcome. This method allows to test for mediation when two exposures share genetic variants as if they had been adjusted for one another^15^.

Here, we used a MVMR study design to investigate the respective causal contributions of adiposity (defined using BMI) and abdominal adiposity (defined using waist circumference and the waist-to-hip ratio adjusted for BMI [WHRadjBMI]) to liver fat accumulation and NAFLD. Second, using a similar strategy, we aimed to determine if abdominal adiposity and liver fat accumulation are independent causal risk factors for T2D and CAD. Taken together, our triangulation of MR methods identify waist circumference as a strong, independent, and causal contributor to NAFLD, type 2 diabetes, and coronary artery disease.

## Methods

### Study populations

Information on the cohorts used in this MR framework is presented in Supplementary Data 1. Briefly, we combined data from publicly accessible GWAS summary statistics of European ancestry in a two-sample MR setting. BMI and waist circumference: The summary statistics of BMI and waist circumference were obtained from the UK Biobank from 461,460 and 462,166 individuals respectively. The GWAS was performed by the MRC IEU open GWAS project^16^. GWAS summary statistics from the GIANT consortium were also included to replicate the estimates obtained with the UK Biobank. These summary statistics for BMI were obtained from a meta-analysis of up to 125 GWAS for 339,224 European individuals^17^. Summary statistics for waist circumference were obtained from a meta-analysis of 232,101 individuals^18^. Measures of BMI and waist circumference were self-reported or measured in a laboratory or in a healthcare setting. Measures were corrected for age, age squared, sex, ancestry-based principal components, and study sites. The resulting residuals were inverse ranked normal transformed with standard deviation (SD) of 1. WHR adjusted for BMI: WHR adjusted for BMI was calculated as the ratio of waist and hip circumferences adjusted for BMI in 485,486 Europeans in the UK Biobank^19^. Measures of WHR and BMI were self-reported, measured in a laboratory or measured in a healthcare setting. Measures of WHRadjBMI were corrected for age, age squared, sex, principal components, and study site. The resulting residuals were transformed to approximate normality with SD of 1 using inverse normal scores. We also included GWAS summary statistics for WHRadjBMI from 210,088 Europeans from the GIANT consortium^18^. In that study, WHRadjBMI was adjusted for age, age-squared, study-specific covariates and then inverse ranked normal transformed prior to genome-wide analysis. NAFLD: We performed a GWAS meta-analysis for clinical diagnosis of NAFLD (8434 cases and 770,180 controls) of European ancestry from four cohorts, as previously described^10^. Briefly, we performed a fixed effect GWAS meta-analysis of The Electronic Medical Records and Genomics (eMERGE) network^20^, the UK Biobank, the Estonian Biobank and FinnGen using the *METAL* package^21^. NAFLD was defined using electronic health record codes or hospital records. Logistic regression analysis was performed with adjustment for age, sex, genotyping site and the first three ancestries-based principal components. Liver Fat: GWAS summary statistics for liver fat were obtained from a GWAS of 32,858 white British participants from the UK Biobank^22^. Magnetic resonance scans were annotated by trained radiologists following a standard procedure. Using this training dataset, deep learning algorithms were then applied to estimate liver fat. The resulting dataset comprises 32,860 liver fat quantification. Liver fat was regressed using BOLT-LMM on gene carrier status, adjusted for genetic sex, age, age^2^, the first 10 principal components of genetic ancestry, scaled scan date, scaled scan time, and study center as fixed effects and genetic relatedness as a random effects term. The resulting residuals were inverse normal transformed prior to GWAS. Coronary artery disease: GWAS summary statistics for CAD were obtained from a GWAS on 122,733 cases and 424,528 controls from CARDIoGRAMplusC4D and UK Biobank^23^. Samples from CARDIoGRAMplusC4D were drawn from a mixed population (Europeans, East Asian, South Asian, Hispanic and African American), with the majority (77%) of the participants from European ancestry. Case status was defined by CAD diagnosis, including myocardial infarction, acute coronary syndrome, chronic stable angina, or coronary stenosis. We also used a different dataset GWAS summary statistics from the CARDIoGRAMplusC4D excluding UK Biobank (60,801 CAD cases and 123,504 controls)^24^. Type 2 diabetes: GWAS summary statistics for type 2 diabetes were obtained from the DIAbetes Genetics Replication and Meta-analysis (DIAGRAM) consortium and UK Biobank (74,124 cases/824,006 controls)^25^. Case status was defined by electronic health records, self-reports, or laboratory derived clinical diagnostics of T2D. We also used a different dataset from the DIAGRAM consortium excluding UK Biobank (26,676 T2D case and 132,532 controls)^26^.

Some of the study samples used to derive our study exposures and outcomes included summary statistics from the UK Biobank, which lead to sample overlap. In univariable MR, sample overlap will bias the estimated results towards the null only when weak instrument is present. In MVMR, the direction of the bias is unclear but will occur only in the presence of weak instrument bias^27^. We included in our primary MR analysis the UK Biobank to increase power and included sensitivity analysis excluding the UK Biobank to remove sample overlap. All GWAS summary statistics were publicly available and accessible through URL. For all included genetic association studies, all participants provided informed consent, and study protocols were approved by their respective local ethical committee. Ethical approval was not required to conduct this study as it only used anonymized GWAS summary statistics.

### Selection of genetic variants and variants harmonization

For univariable MR analysis, we selected all genome-wide significant SNPs (*p*-value < 5e−8). We then ensured the independence of genetic instruments by clumping all neighboring SNPs in a 10 Mb window with a linkage disequilibrium *r*2 < 0.001 using the European 1000-genome LD reference panel. SNPs and relevant association statistics can be found for each exposure in Supplementary Data 2. For multivariable MR analyses, we first extracted all genetic instruments that were previously selected for univariable MR analysis. We then pooled these SNPs to the lowest *p*-value corresponding to any of the exposures, using the same parameter setting as the univariable MR (*r*2 = 0.001 window = 10 Mb). We also included results of two other sensitivity analysis approaches: (1) prioritizing variants with lowest *p* value for BMI; (2) prioritizing SNPs with lowest *p* value for waist circumference. When NAFLD was used as an exposure in MVMR, we pooled the combined list of SNPs by selecting the SNP with the lowest *p*-value for NALFD. This procedure was implemented to select a maximum number of strong genetic instruments, as fewer genetic instruments are available for NAFLD exposure. SNPs in a 2 Mb window of the *HLA*, *ABO,* and *APOE* genetic regions were excluded due to their complex genetic architecture and their widespread pleiotropy (in GRCh37 6:28909037-30913661, 9:135130951-137150617, and 19:44409011-46412650, respectively). Exclusion of pleiotropic genetic regions satisfies the exclusion restriction and the exchangeability assumptions of instrumental variable analyses and strengthen inference of MR analyses. Harmonization was performed by aligning the effect sizes of different studies on the same effect allele. All GWAS summary statistics were reported on the forward strand. When a particular SNP was not present in the outcome datasets, we used a proxy SNPs (*r*2 > 0.6) obtained using linkage disequilibrium matrix of European samples from the 1000 Genomes Project. Instrument strength was quantified using the F-statistic^28^, and the variance explained was quantified using the r2^29^. We calculated *r*2 for each individual SNP. For binary exposures, we calculated *r*2 using equation 10 in Lee et al., 2012^30^ used in the *get_r_from_lor* function in the *TwoSampleMR* package. We calculated the F statistics following the formula ${{F}}=(\frac{{{n}}-{{k}}-1}{{{k}}})(\frac{{{R}}2}{1-{{R}}2})$. Where *n* is the sample size, *k* is the number of instruments used and *R*2 is the sum of the individual *r*2 of each SNP. These statistics can be found in Supplementary Data 3.

### Statistical analyses

For univariable primary MR analysis, we performed the inverse variance weighted (IVW) method with multiplicative random effects with a standard error correction for under dispersion^31^. MR must respect three core assumptions (relevance, independence, and exclusion restriction) for correct causal inference. MR estimates bias occurs if the genetic instruments influence several traits on different causal pathways. This phenomenon, referred to as horizontal pleiotropy, can be balanced by using multiple genetic variants combined with robust univariable MR methods^32^. To verify if pleiotropy likely influenced the primary univariable MR results, we performed 6 different robust MR analyses: MR Egger^33^, the MR-Robust Adjusted Profile Score (MR-RAPS)^34^, the contamination mixture^35^, the weighted median, the weighted mode and the MR-PRESSO^36^, each making a different assumption about the underlying nature of the pleiotropy. Consistent estimates across methods provide further confirmation about the nature of the causal links. All continuous exposure estimates were normalized and reported on a SD scale. For dichotomous traits (i.e., diseased status on NAFLD, T2D and CAD), odds ratios were reported. Univariable MR analyses were performed using the *TwoSampleMR* V.0.5.6 package^37^.

For multivariable primary MR analysis, we conducted the IVW method^38^. The use of MVMR is analogous to the inclusion of measured covariates in a multivariate linear regression. MVMR uses a set of overlapping genetic instrument to estimate the direct effect of an exposure on an outcome. As robust MVMR analyses, we used the multivariable MR-Egger^39^, the multivariable median method, and the multivariable MR-Lasso method^40^. Similar to robust univariable MR analyses, each method makes different assumptions about the underlying nature of the pleiotropy and consistent estimates give confidence in the robustness of the causal findings. Multivariable MR analyses were performed using the *MendelianRandomization* V.0.5.1 package^41^. Conditionnal F-statistics were calculated with formula developed by Sanderson et al., in the *MVMR* V.0.2.0 package^42^. Percentage of mediation was quantified using the formula ($1-\frac{{\theta }_{2}}{{\theta }_{t}}$) Where ${\theta }_{2}$ is the direct effect estimated with IVW-MVMR and ${\theta }_{t}$ is the total effect estimated with univariable IVW-MR^43^.

### Reporting summary

Further information on research design is available in the Nature Research Reporting Summary linked to this article.

## Results

### Causal effect of general and abdominal adiposity to NAFLD and liver fat accumulation

**Fig. 1:**
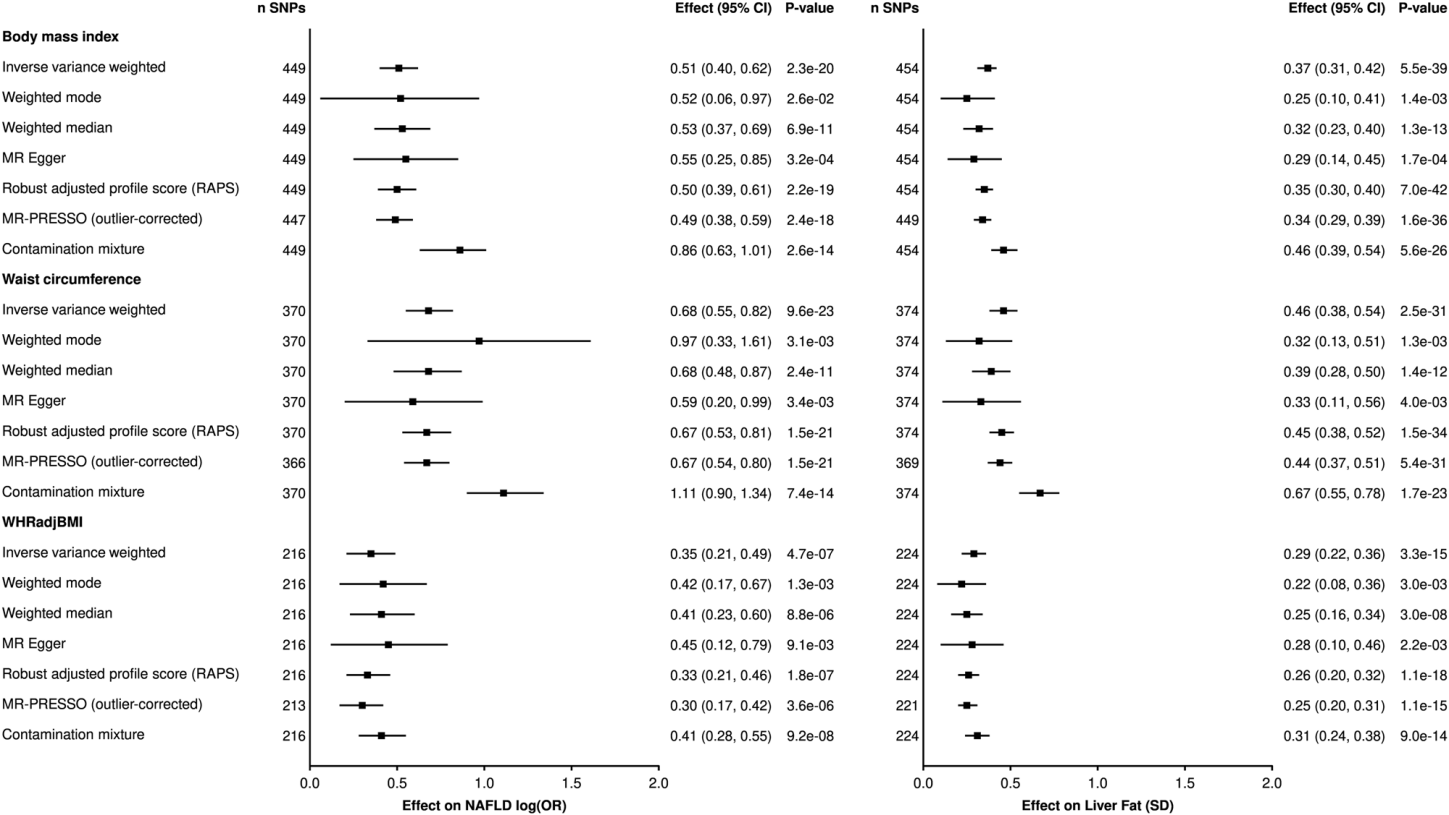
Causal effect of genetically-predicted anthropometric traits on non-alcoholic fatty liver disease (NAFLD) and liver fat accumulation. Inverse-variance weighted Mendelian randomization (IVW-MR) and robust MR analyses were performed to assess the impact of one SD increase body mass index (BMI), waist circumference, and the waist-to-hip ratio adjusted for BMI on NAFLD and liver fat accumulation. Error bars are 95% confidence interval.

We first investigated the causal effect of adiposity (defined by BMI or waist circumference) on NAFLD using Inverse Variance Weighted (IVW)-MR and other robust analyses described in the “Methods” section. Results from all univariable MR methods (Fig. 1 and Supplementary Data 4) including Egger’s intercept (Supplementary Data 5) suggest that BMI and waist circumference are both causally associated with NAFLD. Using 370 SNPs (*r*^2^ = 0.05; F-statistic = 60), a one SD-higher waist circumference had an odds ratio (OR) of 1.98 (95% confidence interval [CI]: 1.73–2.27, *p* = 6.6e−23) for NAFLD. Using 449 SNPs (*r*^2^ = 0.06; F-statistic = 68), a one SD higher BMI had an OR of 1.66 95% CI = 1.49–1.85, *p* = 2.3e−20. Similar associations were found when exposures were derived from the Genetic Investigation of Anthropometric Traits (GIANT) consortium (Supplementary Fig. 1) and when liver fat accumulation was used as the outcome (Fig. 1).

To evaluate the effect of body fat distribution, we investigated the association of WHRadjBMI with NAFLD and liver fat accumulation using multiple univariable MR methods. WHRadjBMI is associated with an elevated waistline and lower BMI. A high WHRadjBMI is a marker of preferential intra-abdominal/visceral adipose tissue accumulation^13^. Using an instrument of 216 SNPs (*r*^2^ = 0.03, F-statistic = 74), a higher genetically predicted WHRadjBMI was associated with NAFLD across all univariable MR methods (OR = 1.42 95% CI = 1.24–1.62, *p* = 4.7e−07) (Fig. 1). Higher genetically predicted WHRadjBMI was also associated with liver fat accumulation across all univariable MR methods. Results were similar when deriving WHRadjBMI from GWAS summary statistics from GIANT. Altogether, these analyses provide evidence that body fat distribution patterns consistent with higher visceral fat accumulation is an important determinant of NAFLD.

**Fig. 2:**
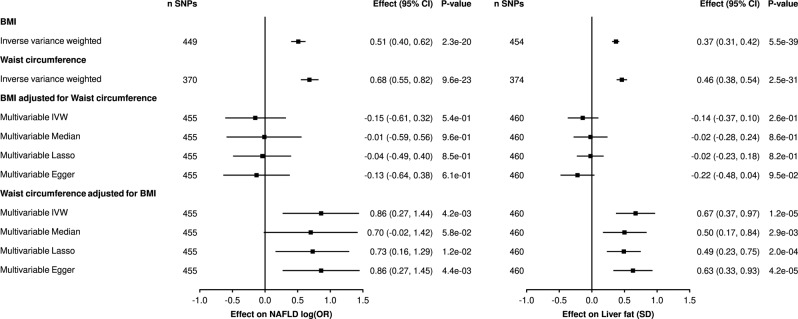
Causal effect genetically predicted waist circumference and body mass index (BMI) on non-alcoholic fatty liver disease (NAFLD) and liver fat accumulation using univariable and multivariable Mendelian randomization. The association between waist circumference and body mass index (per 1-SD increase) and NAFLD and liver fat accumulation is presented using univariable inverse-variance weighted MR and multivariable MR using multiple robust methods. Error bars are 95% confidence interval.

To further investigate the impact of body fat distribution patterns on liver fat accumulation and NAFLD, we evaluated the direct causal effect of abdominal obesity irrespective of general adiposity in a MVMR analysis. BMI and waist circumference shared 114 instruments. When BMI and waist circumference were assessed together in MVMR, only waist circumference (2.35 95% CI = 1.31–4.23, *p* = 4.2e−03) retained a robust association with NAFLD. The effect of BMI on NAFLD upon adjustment for waist circumference was inconclusive (0.86 95% CI = 0.54–1.38, *p* = 5.4e−01) (Fig. 2). Conditional F-statistics for this MVMR analysis were low (1.54 and 1.55 for WC and BMI respectively). However, results were significant and consistent across all robust MVMR analyses and multivariable Egger intercept did not differ from zero indicating that pleiotropy is unlikely to have affected the results (Supplementary Data 6). Similar associations were found when liver fat accumulation was used as the outcome and when using GWAS summary statistics from the GIANT consortium as study exposures for waist circumference and BMI (Supplementary Fig. 2).

**Fig. 3:**
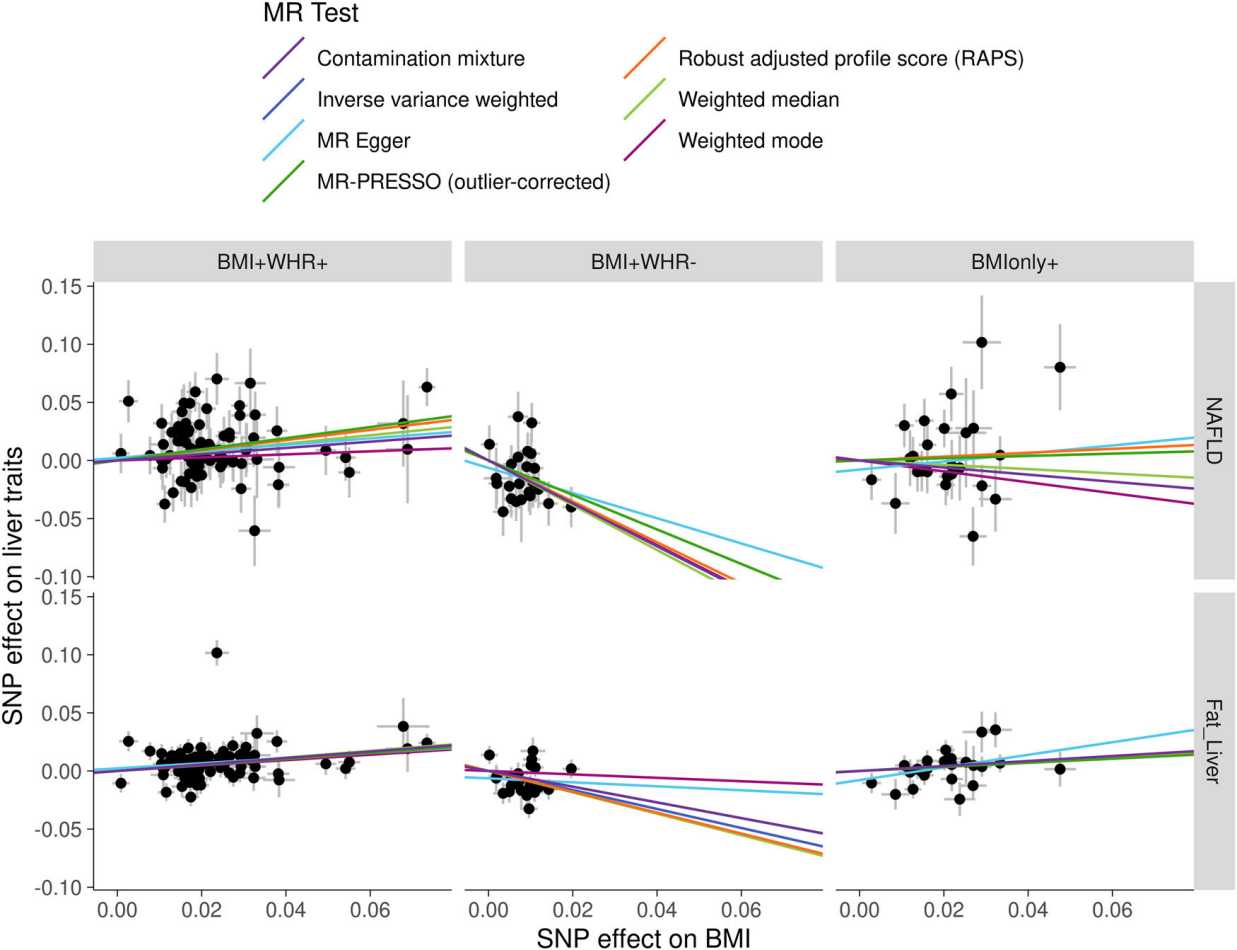
Effect of body mass index (BMI) variants on non-alcoholic fatty liver disease (NAFLD) and liver fat accumulation using group-specific Mendelian randomization. A total of 159 genetic instruments categorized based on their association with BMI and waist-to-hip ratio (WHR): Left panel BMI+WHR+ (nominal significant effects on BMI and WHR with consistent directions), center panel BMI+WHR− (nominal significant effects on BMI and WHR with opposite directions) and right panel BMIonly+ (nominal significant effects on BMI only).

To confirm the impact of body fat distribution indices on liver fat accumulation and NAFLD, we investigated 159 adiposity-related genetic variants derived from ~322,154 subjects from the GIANT consortium^12^. These variants were previously classified into three groups based on the direction of their effects on BMI and WHR: those with positive (*p* < 0.05) association with BMI and positive (*p* < 0.05) association with WHR (BMI+WHR+), negative (*p* < 0.05) association with WHR (BMI+WHR−) or null (*p* > 0.05) association with WHR (BMIonly+)^12^. Group-specific univariable MR using the 80 BMI+WHR+ instruments in the UK Biobank BMI revealed that BMI was positively associated with NALFD risk (OR = 1.61, 95% CI = 1.32–1.98, *p* = 4.3e−06). However, using the 24 BMI+WHR- SNPs, BMI was negatively associated with NAFLD (OR = 0.23, 95% CI = 0.09–0.56, *p* = 1.4e−03). Using the 25 BMIonly+ SNPs the effect of BMI on NAFLD was null (OR = 1.10, 95% CI = 0.66–1.86, *p* = 7.1e−01) (Fig. 3). These results were consistent in robust univariable MR analyses and when evaluating liver fat as the outcome and when using the GIANT consortium as exposure (Supplementary Data 7). Altogether, these results corroborate the univariable and MVMR analyses and provide additional support that intra-abdominal adiposity is a key driver of liver fat accumulation and NAFLD.

### Contributions of abdominal adiposity and liver fat to type 2 diabetes and coronary artery disease

**Fig. 4:**
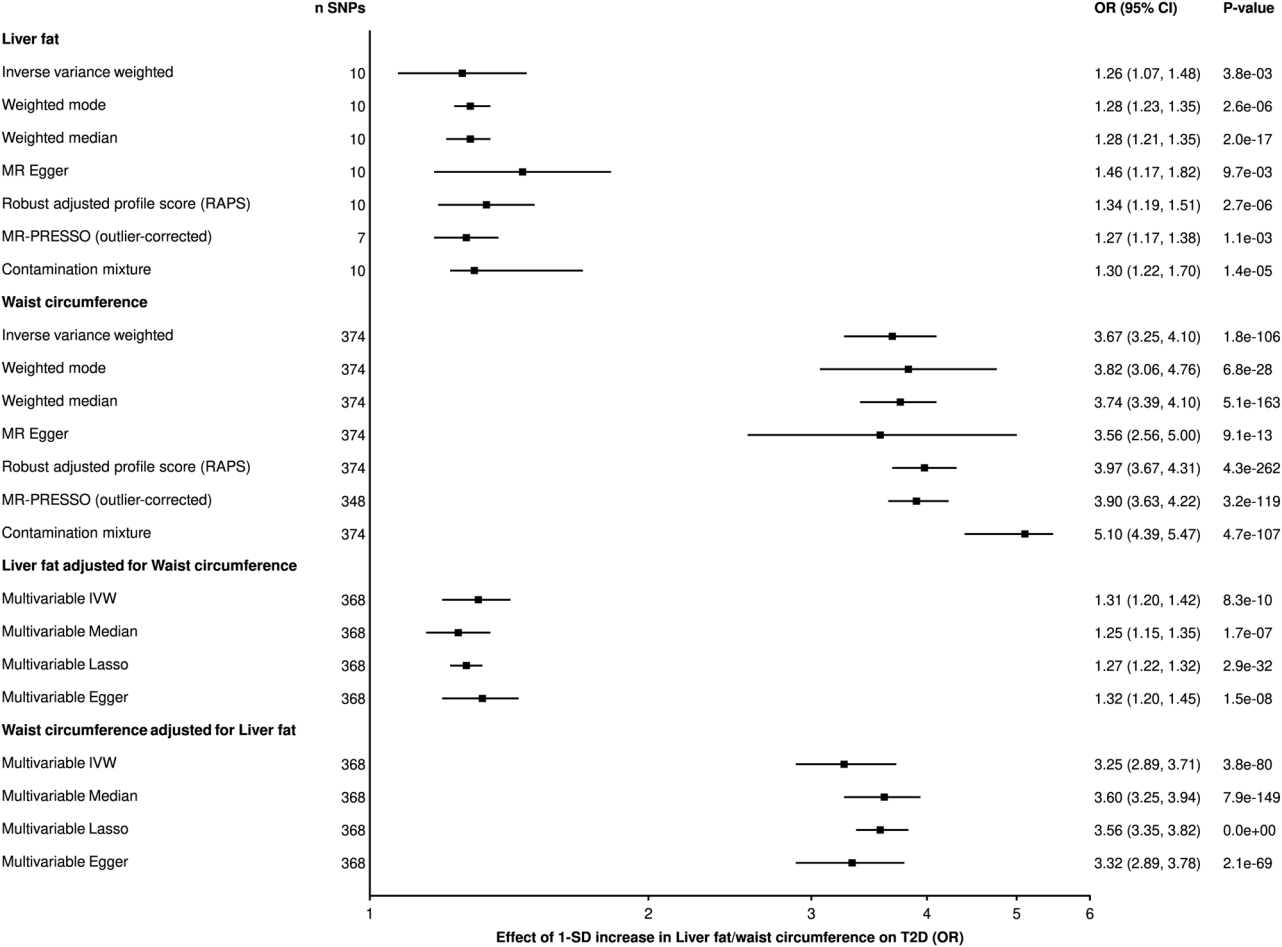
Causal effect of 1-SD increase of waist circumference and liver fat accumulation on type 2 diabetes (T2D) using univariable and multivariable Mendelian randomization (MR). The effect of genetically-predicted waist circumference and liver fat accumulation on T2D using multiple robust MR methods is presented in the top panels and the effect of genetically-predicted waist circumference and liver fat accumulation on T2D using multiple robust multivariable MR methods is presented in the bottom panels. Error bars are 95% confidence interval.

Since abdominal adiposity, NAFLD, T2D, and CAD are highly phenotypically correlated, we next explored the causal effect of abdominal adiposity and NAFLD/liver fat on cardiometabolic diseases. In univariable IVW-MR, using 374 SNPs (*r*^2^ = 0.05; F-statistic = 60), a 1-SD increment in waist circumference increased T2D risk (OR = 3.65 95% CI = 3.25–4.1, *p* =1.8e−106) (Fig. 4) and CAD risk (OR = 1.61 95% CI = 1.5–1.73, *p* = 4.3e−40) (Supplementary Data 4). Using 4 SNPs (*r*^2^ = 0.0005; F-statistic = 2), there was evidence for causal effect of NAFLD on T2D, but not CAD (Supplementary Data 4). Since only four SNPs were associated with NAFLD at the genome-wide significance level (*p* < 5e−8), we investigated the relationship between NAFLD and T2D and CAD using a more lenient threshold (*p* < 5e−6). This analysis confirmed that genetically predicted NAFLD was associated with T2D but not CAD (Supplementary Data 8–9). The binary factor NAFLD, which is diagnosed when liver fat percentage is above 5%, is akin to a dichotomization of the underlying continuous factor “liver fat”. We therefore estimated the causal effect of the continuous variable “liver fat” on cardiometabolic diseases, as recommended^44^. Using 10 SNPs (*r*^2^ = 0.04; F-statistic = 165), a 1-SD increase in liver fat increased the risk of T2D (OR = 1.26 95% CI = 1.08–1.47, *p* = 3.8e−03), but the effect on CAD was inconclusive (OR = 0.90 95% CI = 0.75–1.10, *p* = 3.0e−01) (Fig. 4 and Supplementary Data 3). These causal effects were consistent for all robust univariable MR methods (Fig. 4). Of note, T2D increased liver fat accumulation and NAFLD while there was no evidence for an effect of CAD on liver fat accumulation and NAFLD (Supplementary Data 3).

Since our results have shown that waist circumference increased liver fat and that both traits increased the risk of T2D, we evaluated their respective causal contributions to T2D using MVMR. When waist circumference and liver fat were assessed together in MVMR, waist circumference (OR = 3.27 95% CI = 2.89–3.69, *p* = 3.8e−80) and liver fat (1.31 95% CI = 1.2–1.42, *p* = 8.3e−10) increased the risk of T2D. Results from robust MVMR methods were consistent with a causal effect of both waist circumference and liver fat on T2D (Fig. 4). Mediation analysis suggests that the impact of abdominal adiposity on T2D is 9% mediated by liver fat. In MVMR, the effect of waist circumference on T2D is 4.44 times larger than the effect of liver fat on T2D. The results were similar when deriving waist circumference instruments from GIANT (Supplementary Fig. 3) and when excluding the UK Biobank dataset from the outcome (Supplementary Data 4–5). Results of this analysis revealed that abdominal adiposity is a causal risk factor for CAD and T2D and that the effect of abdominal adiposity on T2D is only modestly (9%) mediated by liver fat.

## Discussion

**Fig. 5:**
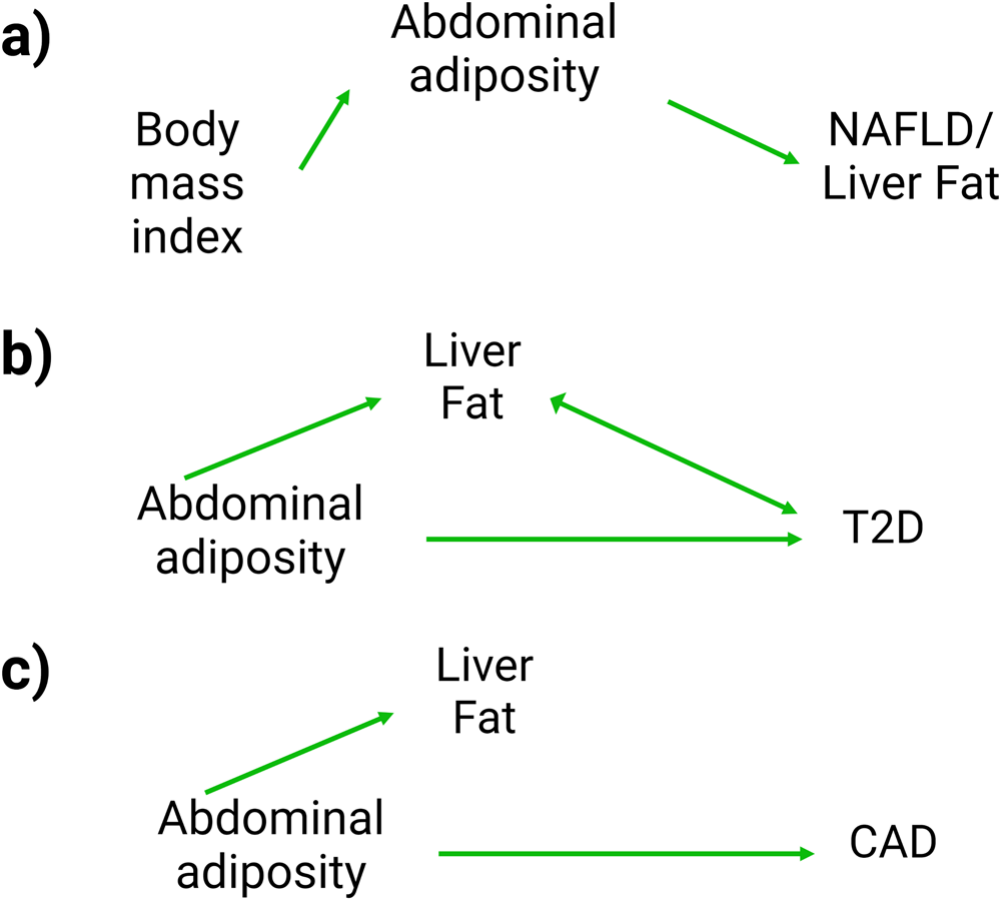
Schematic illustration of the main findings of the study. **a** Both genetically-predicted body mass index and abdominal adiposity are associated with non-alcoholic fatty liver disease (NAFLD) and liver fat accumulation. However, the relationship between genetically-predicted BMI and NAFLD/liver fat accumulation is entirely mediated by genetically-predicted abdominal adiposity. **b** Genetically-predicted abdominal adiposity and NAFLD/liver fat accumulation are both associated with type 2 diabetes (T2D) and their associations with T2D are largely independent from one another. Genetically-predicted T2D is also associated with NAFLD/liver fat accumulation. **c** Genetically-predicted abdominal adiposity, but not genetically-predicted NAFLD/liver fat accumulation is associated with coronary artery disease (CAD). The association between genetically-predicted abdominal adiposity and CAD is not mediated by NAFLD/liver fat accumulation.

In this study, we explored the relationships between general and abdominal adiposity and NAFLD using univariable and multivariable MR. We found that general and abdominal adiposity were causally linked to liver fat accumulation and NAFLD. Results of our multivariable MR analysis suggest that waist circumference is causally linked to liver fat accumulation and NAFLD regardless of BMI, while BMI is not causally linked with NAFLD once waist circumference is taken into account. Having established a causal role of abdominal adiposity on NAFLD and given the results of previous studies linking NAFLD to cardiometabolic diseases such as T2D^45,46^ and CAD^47,48^, we explored whether liver fat accumulation lies in the causal pathway linking abdominal adiposity to T2D and CAD. Using MVMR, our results support that the effect of abdominal adiposity on T2D is substantially larger than the effect of liver fat on T2D. We also showed that the association between abdominal adiposity and CAD is independent of liver fat, thereby highlighting the causal role of abdominal adiposity in the etiology of NAFLD, T2D, and CAD (Fig. 5).

Observational studies have documented similar associations^49^. Liu et al. used bidirectional MR to explore the relationship between NAFLD, adiposity, T2D, and lipid traits^46^. They found that both adiposity and abdominal adiposity had a causal effect on NAFLD. Our study provides additional support for a causal effect of abdominal adiposity on NAFLD using a larger study sample size for the study outcome (NAFLD) and multiple MR methods. Our study is, to our knowledge, the first to report using MVMR a causal association between abdominal adiposity and NAFLD that is independent of the BMI. In their MR investigation, Liu et al. also showed that NAFLD had a causal effect on T2D (OR: 1.3, 95% CI: 1.2, 1.4, *p* = 8.3e−14)^46^. They used 2 genetic instruments for NAFLD, making it impossible to perform pleiotropy robust MR analyses. In our analysis, NAFLD and liver fat was similarly associated with T2D and robust univariable MR analysis were consistent with a causal association. The association was slightly decreased when we accounted for abdominal adiposity using MVMR, suggesting that part of the effect of liver fat on T2D could be attributable to variants influencing primarily abdominal fat accumulation. On the other hand, the point estimate of waist circumference on T2D also only slightly decreased when we accounted for liver fat accumulation, suggesting that the effect of waist circumference on T2D is modestly mediated by liver fat accumulation.

The inability of subcutaneous fat to expand by hyperplasia may partly explain why visceral fat accumulation occurs in genetically predisposed individuals^50^. These excess lipids are then stored in lean tissues such as the liver, heart, and skeletal muscle promoting insulin resistance^4,51^. The mechanisms by which visceral fat contributes to NAFLD may also possibly be explained by the “portal vein theory”^52^. Visceral fat is mostly drained by the portal vein, which delivers its content to the liver and exposes it to high concentrations of free fatty acids and adipokines^53^. These have been hypothesized to lead to metabolic changes in the liver which would ultimately lead to an increased production of VLDL particles, glucose, and inflammatory mediators as well as decreased insulin extraction, potentially leading to T2D and atherosclerosis^50,52^.

From a clinical perspective, results of this study support the idea that previously reported associations between an elevated BMI and NAFLD may be explained by preferential abdominal fat accumulation reflected by higher waist circumference. Indeed, a significant number of individuals with elevated BMI have excess visceral fat increasing their risk of NAFLD^46,54,55^. Our results also underline the limitations of the sole use of BMI in clinical practice to assess the risk associated with obesity/ectopic fat distribution. The failure of BMI to capture cardiometabolic risk had already been suggested by observational and MR studies^4,5,56^. Our study adds evidence supporting waist circumference as a simple tool to assess obesity-related health hazards.

These results should encourage clinical interventions focused on visceral fat reduction, not only overall body weight reduction, to prevent cardiometabolic diseases such as NAFLD, T2D and CAD. Visceral fat can be targeted with physical activity and dietary interventions even in the absence of weight loss. A weight loss of about 5% can result in a 15–25% visceral fat reduction^57^. The Mediterranean diet as well as diets lower in fat and/or carbohydrate may be effective ways of reducing visceral fat, especially in physically active individuals^4,58,59^. There is also evidence that thiazolidinediones (TZDs) such as pioglitazone and rosiglitazone, used in the treatment of T2D, increase subcutaneous adipocytes’ storage capacity and lower T2D risk^60^. Results of the VICTORY trial, a study aimed at assessing the safety and efficiency of rosiglitazone on saphenous vein graft atherosclerosis and the cardiometabolic risk profile, showed that rosiglitazone treatment induced a 3 kg weight gain over 12 months and no change in visceral adiposity^61^. Pioglitazone has also been shown to reduce hepatic steatosis and inflammation in patients with NASH^62^ thereby providing randomized clinical trial support to our MR findings. Semaglutide, a glucagon-like protein-1 (GLP-1) receptor agonist, has recently been shown to increase the rate of NASH resolution compared with placebo^63^. Recent studies on another GLP-1 receptor agonist liraglutide and a dual glucose-dependent insulinotropic polypeptide (GIP) and GLP-1 receptor agonist also recently provided evidence that this pathway may induce a preferential loss in visceral adipose tissue and liver fat accumulation^57,64^.

An important strength of the current study is the use of the largest liver fat accumulation and NAFLD datasets available to date. Additionally, the use of MVMR enabled the estimation of the direct effect of closely related risk factors on cardiometabolic outcomes while mitigating bias from confounding and reverse causality compared to classic observational studies. Our study, however, has limitations. The NAFLD GWAS included ~8000 cases and ~750,000 controls, but the population prevalence of NAFLD has been estimated to 25%. Hence, it is probable that some controls could have been misclassified. While it is important to acknowledge this limitation, we believe that such misclassification could bias our results towards the null and underestimate the strength of the reported associations. These associations were also consistent when using liver fat accumulation measured in 32,858 individuals, which better represents population prevalence for this trait compared with NAFLD. In contrast to adiposity-related traits, few genetic instruments were available for NAFLD and liver fat when these traits were used as exposures, making the assessment of pleiotropy more challenging. Consequently, we used a more lenient *p*-value threshold when NAFLD was used as the study exposure, increasing variance explained with the drawback of having more chance of including invalid or pleiotropic instruments. Another potential limitation to this work is that a binary trait (NAFLD) was used as an exposure. This could have led to the violation of the exchangeability assumption^44^. For this reason, we only tested the causal null hypothesis, instead of attempting to calculate the causal estimate. We also used the underlying continuous risk factor “liver fat accumulation” to estimate causal estimates. Finally, although the instrument strength was adequate to perform univariable MR analyses, waist circumference and BMI had low conditional F-statistics in MVMR, making these instruments vulnerable to weak instrument bias. Robust MR analyses and egger intercept indicated that other assumptions were likely to be satisfied.

In conclusion, results of this MVMR investigation suggest that independently of BMI, waist circumference is a strong and causal contributor to NAFLD. Also, the association between waist circumference and T2D and CAD is largely independent of liver fat. Altogether, the results are consistent with the hypothesis that abdominal adiposity may represent a root cause of cardiometabolic diseases. Clinical interventions targeting ectopic lipid deposition may be the key to the treatment of cardiometabolic diseases such as NAFLD, CAD, and T2D.

### Institutional review board approval

All GWAS summary statistics were publicly available and accessible through URL. For all included genetic association studies, all participants provided informed consent and study protocols were approved by their respective local ethical committee.

## Supplementary information

Peer Review File

Supplementary Information

Description of Additional Supplementary Files

Supplementary Data 1

Supplementary Data 2

Supplementary Data 3

Supplementary Data 4

Supplementary Data 5

Supplementary Data 6

Supplementary Data 7

Supplementary Data 8

Supplementary Data 9

Reporting Summary

## Data Availability

Source data and GWAS summary statistics can be found following the following links: GWAS summary statistics for anthropometric traits from GIANT are available at: https://portals.broadinstitute.org/collaboration/giant/index.php/GIANT_consortium_data_files GWAS summary statistics for BMI from UKB are available via the MR Base GWAS catalog at id “ukb-b-19953”. GWAS summary statistics for waist circumference from UKB are available via the MR Base GWAS catalog at id “ukb-b-9405”. GWAS summary statistics for T2D are available at: http://diagramconsortium.org/downloads.html GWAS summary statistics for CAD are available at: https://www.cardiomics.net/download-data. GWAS summary statistics for NAFLD are available at: https://www.ebi.ac.uk/gwas/studies/GCST90091033. We make accessible a small subset of these summary statistics to reproduce the figures and the results on our GitHub^65^.

## Code availability

The code used to perform the analysis can be found on GitHub^65^. The *TwoSampleMR* package is available at: https://github.com/MRCIEU/TwoSampleMR The *MendelianRandomization* package is available at: https://github.com/cran/MendelianRandomization The *data.table* package is available at https://github.com/Rdatatable/data.table The *tidyverse* package collection is available at: https://github.com/tidyverse/tidyverse.

## References

[1] Chalasani N (2018). The diagnosis and management of nonalcoholic fatty liver disease: Practice guidance from the American Association for the Study of Liver Diseases. Hepatology.

[2] Charlton MR (2011). Frequency and outcomes of liver transplantation for nonalcoholic steatohepatitis in the United States. Gastroenterology.

[3] Younossi ZM (2016). Global epidemiology of nonalcoholic fatty liver disease-Meta-analytic assessment of prevalence, incidence, and outcomes. Hepatology.

[4] Ross R (2020). Waist circumference as a vital sign in clinical practice: A consensus statement from the IAS and ICCR working group on visceral obesity. Nat. Rev. Endocrinol..

[5] Nazare J-A (2015). Usefulness of measuring both body mass index and waist circumference for the estimation of visceral adiposity and related cardiometabolic risk profile (from the INSPIRE ME IAA study). Am. J. Cardiol..

[6] Fabbrini E (2009). Intrahepatic fat, not visceral fat, is linked with metabolic complications of obesity. Proc. Natl Acad. Sci. USA.

[7] Kotronen A, Yki-Järvinen H (2008). Fatty liver: A novel component of the metabolic syndrome. Arterioscler. Thromb. Vasc. Biol..

[8] Ndumele CE (2011). Hepatic steatosis, obesity, and the metabolic syndrome are independently and additively associated with increased systemic inflammation. Arterioscler. Thromb. Vasc. Biol..

[9] Tilg H, Moschen AR, Roden M (2017). NAFLD and diabetes mellitus. Nat. Rev. Gastroenterol. Hepatol..

[10] Ghodsian N (2021). Electronic health record-based genome-wide meta-analysis provides insights on the genetic architecture of non-alcoholic fatty liver disease. Cell Rep. Med..

[11] Smith GD, Ebrahim S (2003). ‘Mendelian randomization’: Can genetic epidemiology contribute to understanding environmental determinants of disease?. Int. J. Epidemiol..

[12] Winkler TW (2018). A joint view on genetic variants for adiposity differentiates subtypes with distinct metabolic implications. Nat. Commun..

[13] Emdin CA (2017). Genetic association of waist-to-hip ratio with cardiometabolic traits, type 2 diabetes, and coronary heart disease. JAMA.

[14] Welsh P (2010). Unraveling the directional link between adiposity and inflammation: A bidirectional Mendelian randomization approach. J. Clin. Endocrinol. Metab..

[15] Sanderson E, Davey Smith G, Windmeijer F, Bowden J (2019). An examination of multivariable Mendelian randomization in the single-sample and two-sample summary data settings. Int. J. Epidemiol..

[16] Elsworth, B. et al. The MRC IEU OpenGWAS data infrastructure. Preprint at 10.1101/2020.08.10.244293 (2020).

[17] Locke AE (2015). Genetic studies of body mass index yield new insights for obesity biology. Nature.

[18] Shungin D (2015). New genetic loci link adipose and insulin biology to body fat distribution. Nature.

[19] Pulit SL (2019). Meta-analysis of genome-wide association studies for body fat distribution in 694 649 individuals of European ancestry. Hum. Mol. Genet..

[20] Namjou B (2019). GWAS and enrichment analyses of non-alcoholic fatty liver disease identify new trait-associated genes and pathways across eMERGE Network. BMC Med..

[21] Willer CJ, Li Y, Abecasis GR (2010). METAL: Fast and efficient meta-analysis of genomewide association scans. Bioinformatics.

[22] Liu Y (2021). Genetic architecture of 11 organ traits derived from abdominal MRI using deep learning. eLife.

[23] van der Harst P, Verweij N (2018). Identification of 64 novel genetic loci provides an expanded view on the genetic architecture of coronary artery disease. Circ. Res..

[24] Nikpay M (2015). A comprehensive 1000 genomes-based genome-wide association meta-analysis of coronary artery disease. Nat. Genet..

[25] Mahajan A (2018). Fine-mapping type 2 diabetes loci to single-variant resolution using high-density imputation and islet-specific epigenome maps. Nat. Genet..

[26] Scott RA (2017). An expanded genome-wide association study of type 2 diabetes in Europeans. Diabetes.

[27] Sanderson E, Windmeijer F (2016). A weak instrument F-test in linear IV models with multiple endogenous variables. J. Econometrics.

[28] Burgess S, Thompson SG, CRP CHD Genetics Collaboration. (2011). Avoiding bias from weak instruments in Mendelian randomization studies. Int. J. Epidemiol..

[29] Pierce BL, Ahsan H, VanderWeele TJ (2011). Power and instrument strength requirements for Mendelian randomization studies using multiple genetic variants. Int. J. Epidemiol..

[30] Lee SH, Goddard ME, Wray NR, Visscher PM (2012). A better coefficient of determination for genetic profile analysis. Genetic Epidemiol..

[31] Burgess S, Foley CN, Zuber V (2018). Inferring causal relationships between risk factors and outcomes from genome-wide association study data. Annu. Rev. Genomics Hum. Genet..

[32] Slob EAW, Burgess S (2020). A comparison of robust Mendelian randomization methods using summary data. Genet Epidemiol..

[33] Bowden J, Davey Smith G, Burgess S (2015). Mendelian randomization with invalid instruments: Effect estimation and bias detection through Egger regression. Int. J. Epidemiol..

[34] Zhao, Q., Wang, J., Hemani, G., Bowden, J. & Small, D. S. Statistical inference in two-sample summary-data Mendelian randomization using robust adjusted profile score. Preprint at 10.48550/arXiv.1801.09652 (2018).

[35] Burgess S, Foley CN, Allara E, Staley JR, Howson JMM (2020). A robust and efficient method for Mendelian randomization with hundreds of genetic variants. Nat. Commun..

[36] Verbanck M, Chen C-Y, Neale B, Do R (2018). Detection of widespread horizontal pleiotropy in causal relationships inferred from Mendelian randomization between complex traits and diseases. Nat. Genet..

[37] Hemani G (2018). The MR-Base platform supports systematic causal inference across the human phenome. eLife.

[38] Burgess S, Thompson SG (2015). Multivariable Mendelian randomization: The use of pleiotropic genetic variants to estimate causal effects. Am. J. Epidemiol..

[39] Rees, J. M. B., Wood, A. & Burgess, S. Extending the MR-Egger method for multivariable Mendelian randomization to correct for both measured and unmeasured pleiotropy. *Stat Med*. **36**, 4705–4718 (2017). 10.1002/sim.7492 PMC5725762 28960498

[40] Grant, A. J. & Burgess, S. Pleiotropy robust methods for multivariable Mendelian randomization. *Stat. Med.*10.1002/sim.9156 (2021). 10.1002/sim.9156 PMC7612169 34342032

[41] Yavorska OO, Burgess S (2017). MendelianRandomization: an R package for performing Mendelian randomization analyses using summarized data. Int. J. Epidemiol..

[42] Sanderson E, Spiller W, Bowden J (2021). Testing and correcting for weak and pleiotropic instruments in two-sample multivariable Mendelian randomization. Stat. Med..

[43] Burgess S (2017). Dissecting causal pathways using Mendelian randomization with summarized genetic data: Application to age at menarche and risk of breast cancer. Genetics.

[44] Burgess S, Labrecque JA (2018). Mendelian randomization with a binary exposure variable: Interpretation and presentation of causal estimates. Eur. J. Epidemiol..

[45] Marott SCW, Nordestgaard BG, Tybjærg-Hansen A, Benn M (2016). Components of the metabolic syndrome and risk of type 2 diabetes. J. Clin. Endocrinol. Metab..

[46] Liu Z (2020). Causal relationships between NAFLD, T2D, and obesity have implications for disease subphenotyping. J. Hepatol..

[47] Lauridsen BK (2018). Liver fat content, non-alcoholic fatty liver disease, and ischaemic heart disease: Mendelian randomization and meta-analysis of 279,013 individuals. Eur. Heart J..

[48] Zhang X (2018). Assessing causal estimates of the association of obesity-related traits with coronary artery disease using a Mendelian randomization approach. Sci. Rep..

[49] Jarvis H (2020). Metabolic risk factors and incident advanced liver disease in non-alcoholic fatty liver disease (NAFLD): A systematic review and meta-analysis of population-based observational studies. PLoS Med..

[50] Tchernof A, Després J-P (2013). Pathophysiology of human visceral obesity: An update. Physiol. Rev..

[51] Ye, R. Z., Richard, G., Gévry, N., Tchernof, A. & Carpentier, A. C. Fat cell size: Measurement methods, pathophysiological origins, and relationships with metabolic dysregulations. *Endocrine Rev.*10.1210/endrev/bnab018 (2021). 10.1210/endrev/bnab018 PMC8755996 34100954

[52] Rytka JM, Wueest S, Schoenle EJ, Konrad D (2011). The portal theory supported by venous drainage–selective fat transplantation. Diabetes.

[53] Item F, Konrad D (2012). Visceral fat and metabolic inflammation: The portal theory revisited. Obesity Rev..

[54] Loomis AK (2016). Body mass index and risk of nonalcoholic fatty liver disease: Two electronic health record prospective studies. J. Clin. Endocrinol. Metab..

[55] Miyake T (2013). Body mass index is the most useful predictive factor for the onset of nonalcoholic fatty liver disease: a community-based retrospective longitudinal cohort study. J. Gastroenterol..

[56] Snijder MB, van Dam RM, Visser M, Seidell JC (2006). What aspects of body fat are particularly hazardous and how do we measure them?. Int. J. Epidemiol..

[57] Neeland IJ (2019). Visceral and ectopic fat, atherosclerosis, and cardiometabolic disease: A position statement. Lancet Diabetes Endocrinol..

[58] Gepner Y (2018). Effect of distinct lifestyle interventions on mobilization of fat storage pools: CENTRAL magnetic resonance imaging randomized controlled trial. Circulation.

[59] Verheggen RJHM (2016). A systematic review and meta-analysis on the effects of exercise training versus hypocaloric diet: Distinct effects on body weight and visceral adipose tissue. Obes. Rev..

[60] Unger RH (2008). Reinventing type 2 diabetes: Pathogenesis, treatment, and prevention. JAMA.

[61] Bertrand OF (2010). Cardiometabolic effects of rosiglitazone in patients with type 2 diabetes and coronary artery bypass grafts: A randomized placebo-controlled clinical trial. Atherosclerosis.

[62] Sanyal AJ (2010). Pioglitazone, vitamin E, or placebo for nonalcoholic steatohepatitis. N. Engl. J. Med..

[63] Newsome PN (2021). A placebo-controlled trial of subcutaneous semaglutide in nonalcoholic steatohepatitis. N. Engl. J. Med..

[64] Gastaldelli, A. et al. Effect of tirzepatide versus insulin degludec on liver fat content and abdominal adipose tissue in people with type 2 diabetes (SURPASS-3 MRI): a substudy of the randomised, open-label, parallel-group, phase 3 SURPASS-3 trial. *Lancet Diabetes Endocrinol.*10.1016/S2213-8587(22)00070-5 (2022). 10.1016/S2213-8587(22)00070-5 35468325

[65] Gagnon, É. & LaboArsenault. LaboArsenault/BMI_WC_NAFLD: CommsMed v1.0. 10.5281/zenodo.7116676 (2022).

