# Supplementary material for: Mendelian randomization prioritizes abdominal adiposity as an independent causal factor for liver fat accumulation and cardiometabolic diseases
Source: Commun Med (Lond). 2022 Oct 13;2:130. doi: 10.1038/s43856-022-00196-3 (PMC9561122; doi:10.1038/s43856-022-00196-3)
Supplement: Supplementary file 2 — Supplementary Information [file 43856_2022_196_MOESM2_ESM.pdf]

Supplementary Materials

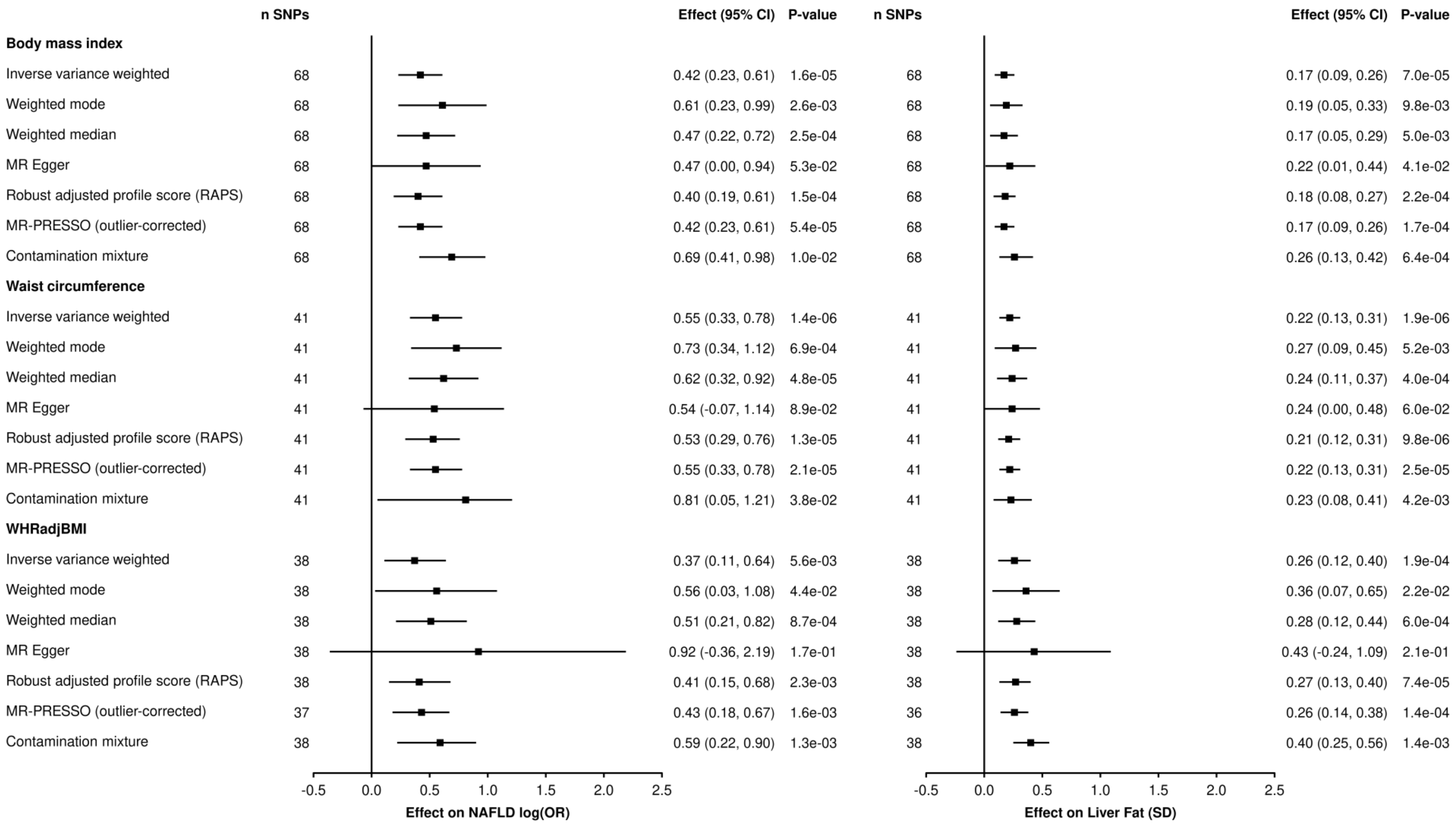

Supplementary Figure 1. Causal effect of adiposity indices from GIANT on non-alcoholic fatty liver disease (NAFLD) and liver fat using inverse-variance weighted Mendelian randomization (IVW-MR) and robust MR analyses.

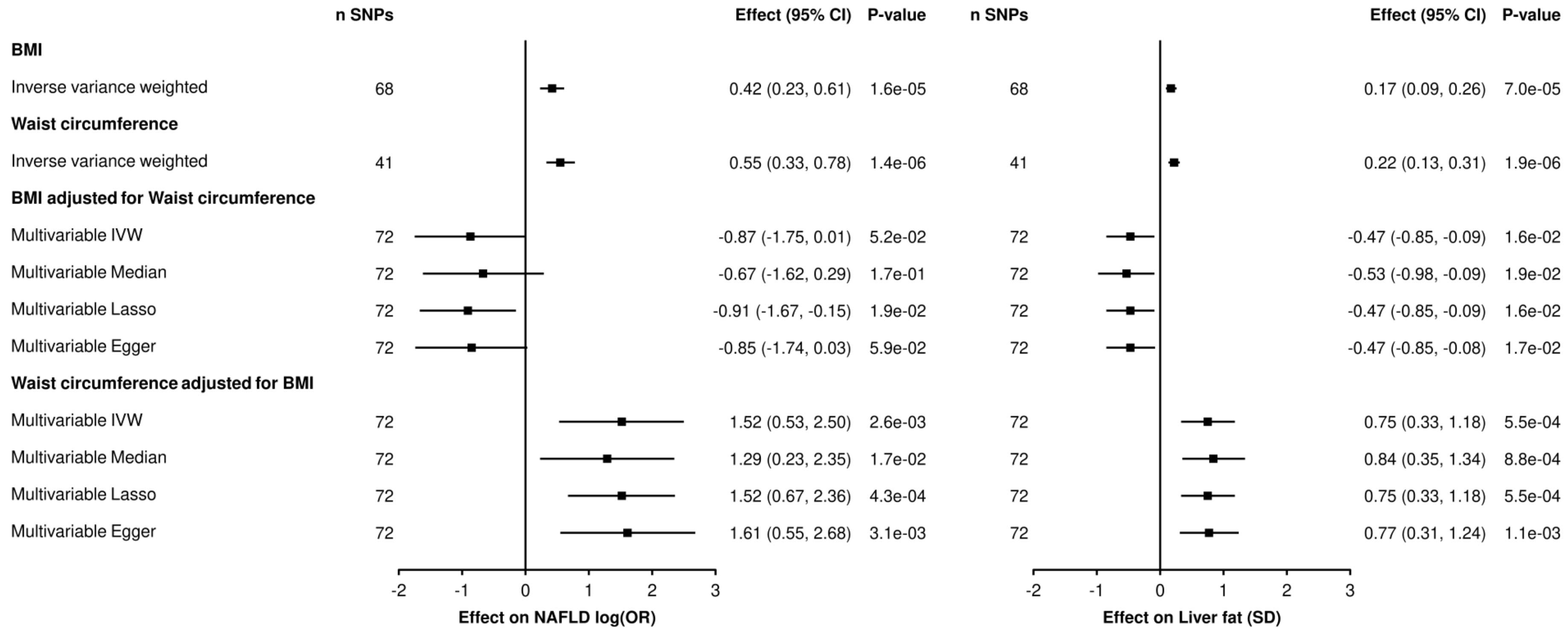

Supplementary Figure 2. Association between waist circumference and body mass index from GIANT with non-alcoholic fattyliver disease and liver fat using univariable and multivariable Mendelian randomization.

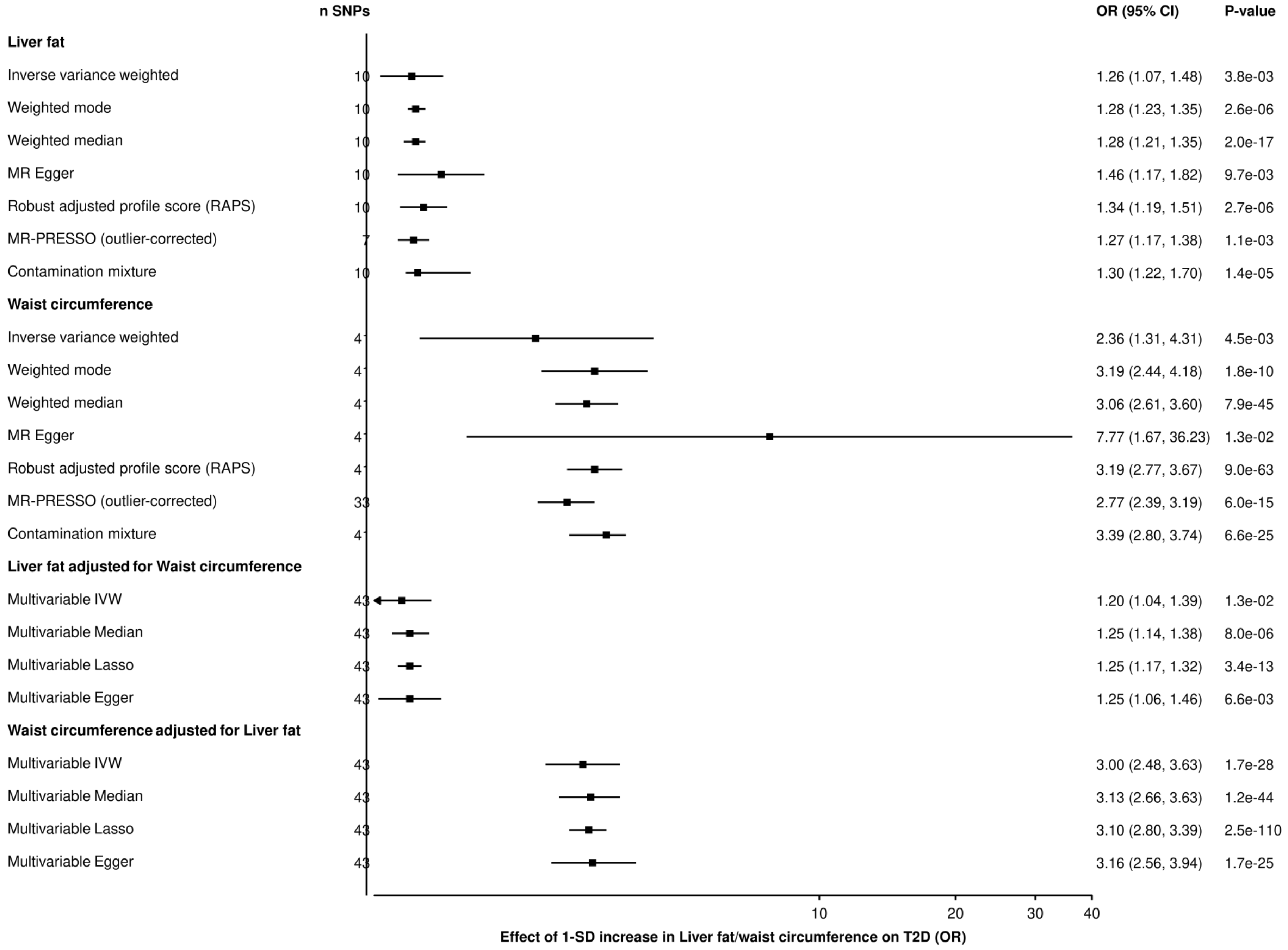
n SNPs
OR (95% CI)
P-value
Liver fat
Inverse variance weighted
10
1.26 (1.07, 1.48)
3.8e-03
Weighted mode
10
1.28 (1.23, 1.35)
2.6e-06
Weighted median
10
1.28 (1.21, 1.35)
2.0e-17
MR Egger
10
1.46 (1.17, 1.82)
9.7e-03
Robust adjusted profile score (RAPS)
10
1.34 (1.19, 1.51)
2.7e-06
MR-PRESSO (outlier-corrected)
7
1.27 (1.17, 1.38)
1.1e-03
Contamination mixture
10
1.30 (1.22, 1.70)
1.4e-05
Waist circumference
Inverse variance weighted
41
2.36 (1.31, 4.31)
4.5e-03
Weighted mode
41
3.19 (2.44, 4.18)
1.8e-10
Weighted median
41
3.06 (2.61, 3.60)
7.9e-45
MR Egger
41
7.77 (1.67, 36.23)
1.3e-02
Robust adjusted profile score (RAPS)
41
3.19 (2.77, 3.67)
9.0e-63
MR-PRESSO (outlier-corrected)
33
2.77 (2.39, 3.19)
6.0e-15
Contamination mixture
41
3.39 (2.80, 3.74)
6.6e-25
Liver fat adjusted for Waist circumference
Multivariable IVW
43
1.20 (1.04, 1.39)
1.3e-02
Multivariable Median
43
1.25 (1.14, 1.38)
8.0e-06
Multivariable Lasso
43
1.25 (1.17, 1.32)
3.4e-13
Multivariable Egger
43
1.25 (1.06, 1.46)
6.6e-03
Waist circumference adjusted for Liver fat
Multivariable IVW
43
3.00 (2.48, 3.63)
1.7e-28
Multivariable Median
43
3.13 (2.66, 3.63)
1.2e-44
Multivariable Lasso
43
3.10 (2.80, 3.39)
2.5e-110
Multivariable Egger
43
3.16 (2.56, 3.94)
1.7e-25
10
20
30
40
Effect of 1-SD increase in Liver fat/waist circumference on T2D (OR)

Supplementary Figure 3. Effect of liver fat and waist circumference from GIANT on type 2 diabetes using univariable and multivariable Mendelian randomization.
