# Supplementary material for: Mendelian randomization prioritizes abdominal adiposity as an independent causal factor for liver fat accumulation and cardiometabolic diseases
Source: Commun Med (Lond). 2022 Oct 13;2:130. doi: 10.1038/s43856-022-00196-3 (PMC9561122; doi:10.1038/s43856-022-00196-3)
Supplement: Supplementary file 3 — Description of Additional Supplementary Files [file 43856_2022_196_MOESM3_ESM.pdf]

Description of Additional Supplementary Files

File Name: Supplementary Data 1

Description: Description of the datasets used.

File Name: Supplementary Data 2

Description: Harmonised data sets.

File Name: Supplementary Data 3

Description: Univariable Mendelian Randomization results

File Name: Supplementary Data 4

Description: Univariable Mendelian Randomization Egger's intercept

File Name: Supplementary Data 5

Description: Multivariable Mendelian randomization results

File Name: Supplementary Data 6

Description: Group specific Mendelian randomization results

File Name: Supplementary Data 7

Description: NAFLD effect on T2D and CAD using p-value threshold of 5e-6 and LD clump of $R2<0.001$

File Name: Supplementary Data 8

Description: NAFLD egger intercept on T2D and CAD using p-value threshold of 5e-6 and LD clump of $R2<0.001$

File Name: Supplementary Data 9

Description: Instrument strength and heterogeneity statistics for univariable MR
