# Supplementary material for: Mendelian randomization prioritizes abdominal adiposity as an independent causal factor for liver fat accumulation and cardiometabolic diseases
Source: Commun Med (Lond). 2022 Oct 13;2:130. doi: 10.1038/s43856-022-00196-3 (PMC9561122; doi:10.1038/s43856-022-00196-3)
Supplement: Supplementary file 13 — Reporting Summary [file 43856_2022_196_MOESM13_ESM.pdf]

nature research

Corresponding author(s): *Double-blind peer review submissions: write DBPR and your manuscript number here instead of author names.*

Last updated by author(s): *YYYY-MM-DD*

Reporting Summary

Nature Research wishes to improve the reproducibility of the work that we publish. This form provides structure for consistency and transparency in reporting. For further information on Nature Research policies, see our Editorial Policies and the Editorial Policy Checklist.

Statistics

For all statistical analyses, confirm that the following items are present in the figure legend, table legend, main text, or Methods section.

n/a	Confirmed	
☐	☒	The exact sample size (*n*) for each experimental group/condition, given as a discrete number and unit of measurement
☐	☒	A statement on whether measurements were taken from distinct samples or whether the same sample was measured repeatedly
☐	☒	The statistical test(s) used AND whether they are one- or two-sided<br>*Only common tests should be described solely by name; describe more complex techniques in the Methods section.*
☐	☒	A description of all covariates tested
☐	☒	A description of any assumptions or corrections, such as tests of normality and adjustment for multiple comparisons
☐	☒	A full description of the statistical parameters including central tendency (e.g. means) or other basic estimates (e.g. regression coefficient) AND variation (e.g. standard deviation) or associated estimates of uncertainty (e.g. confidence intervals)
☐	☒	For null hypothesis testing, the test statistic (e.g. *F*, *t*, *r*) with confidence intervals, effect sizes, degrees of freedom and *P* value noted<br>*Give P values as exact values whenever suitable.*
☒	☐	For Bayesian analysis, information on the choice of priors and Markov chain Monte Carlo settings
☒	☐	For hierarchical and complex designs, identification of the appropriate level for tests and full reporting of outcomes
☒	☐	Estimates of effect sizes (e.g. Cohen's *d*, Pearson's *r*), indicating how they were calculated

Our web collection on statistics for biologists contains articles on many of the points above.

Software and code

Policy information about availability of computer code

Data collection	*Provide a description of all commercial, open source and custom code used to collect the data in this study, specifying the version used OR state that no software was used.*
Data analysis	The code used to perform the analysis can be found on https://github.com/LaboArsenault/BMI_WC_NAFLD Univariable MR analyses were performed using the TwoSampleMR V.0.5.6 package (Hemani et al. 2018). . Multivariable MR analyses were performed using the MendelianRandomization V.0.5.1 package (Yavorska and Burgess 2017).

For manuscripts utilizing custom algorithms or software that are central to the research but not yet described in published literature, software must be made available to editors and reviewers. We strongly encourage code deposition in a community repository (e.g. GitHub). See the Nature Research guidelines for submitting code & software for further information.

Data

Policy information about availability of data

All manuscripts must include a data availability statement. This statement should provide the following information, where applicable:

- Accession codes, unique identifiers, or web links for publicly available datasets
- A list of figures that have associated raw data
- A description of any restrictions on data availability

GWAS summary statistics for anthropometric traits from GIANT are available at: https://portals.broadinstitute.org/collaboration/giant/index.php/GIANT_consortium_data_files
GWAS summary statistics for BMI from UKB are available via the MR Base GWAS catalogue at id "ukb-b-19953".
GWAS summary statistics for waist circumference from UKB are available via the MR Base GWAS catalogue at id "ukb-b-9405".
GWAS summary statistics for T2D are available at: http://diagramconsortium.org/downloads.html

GWAS summary statistics for CAD are available at: https://www.cardiomics.net/download-data
GWAS summary statistics for NAFLD are available at: https://www.ebi.ac.uk/gwas/studies/GCST90091033

Field-specific reporting

Please select the one below that is the best fit for your research. If you are not sure, read the appropriate sections before making your selection.

☒ Life sciences ☐ Behavioural & social sciences ☐ Ecological, evolutionary & environmental sciences

For a reference copy of the document with all sections, see nature.com/documents/nr-reporting-summary-flat.pdf

Life sciences study design

All studies must disclose on these points even when the disclosure is negative.

Sample size	Analysis were performed exclusively on publically available GWAS summary statistics.
Data exclusions	No data were excluded from the analysis.
Replication	We performed replication analysis when feasible using non-overlapping samples.
Randomization	Randomization was not relevant in the study as Mendelian randomization draw on observational data.
Blinding	No experiments were performed. Therefore blinding was irrelevant.

Reporting for specific materials, systems and methods

We require information from authors about some types of materials, experimental systems and methods used in many studies. Here, indicate whether each material, system or method listed is relevant to your study. If you are not sure if a list item applies to your research, read the appropriate section before selecting a response.

Materials & experimental systems

n/a	Involved in the study	
☒	☐	Antibodies
☒	☐	Eukaryotic cell lines
☒	☐	Palaeontology and archaeology
☒	☐	Animals and other organisms
☒	☐	Human research participants
☒	☐	Clinical data
☒	☐	Dual use research of concern

Methods

n/a	Involved in the study	
☒	☐	ChIP-seq
☒	☐	Flow cytometry
☒	☐	MRI-based neuroimaging
